# A study exploring critical pathways in clear cell renal cell carcinoma

**DOI:** 10.3892/etm.2013.1392

**Published:** 2013-11-07

**Authors:** ZISAN ZENG, TENGCHENG QUE, JIANGE ZHANG, YANLING HU

**Affiliations:** 1 Department of Radiology, The First Affiliated Hospital of Guangxi Medical University, Guangxi 530021, P.R. China; 2 Wild Animal Rescue Center, Forestry Bureau of Guangxi, Guangxi 530021, P.R. China; 3 Department of Urology, The First Affiliated Hospital of Guangxi Medical University, Guangxi 530021, P.R. China; 4 Medical Scientific Research Center, Guangxi Medical University, Guangxi 530021, P.R. China; 5 Guangxi Medical University, Nanning, Guangxi 530021, P.R. China

**Keywords:** clear cell renal cell carcinoma, pathway analysis, meta-analyis, gene expression

## Abstract

Renal cell carcinoma (RCC) is the most lethal type of cancer in the urinary system and often presents as a metastatic disease. Furthermore, there are no effective treatments for the disease. Several studies based on gene expression profiling have been performed with the aim of gaining insights into the pathogenesis of RCC; however, few studies have investigated RCC at the pathway level to search for the possible pathways involved in clear cell RCC (CCRCC). In this study, gene set enrichment analysis (GSEA) was conducted on microarray datasets from CCRCC tissue. DAVID functional enrichment analysis was performed based on the dysregulated genes that were identified in a meta-analysis performed on the microarray datasets from CCRCC tissue. In GSEA, 17 down- and 12 upregulated pathways coexisted in six datasets. The majority of the upregulated pathways were associated with the immune system. In addition, 32 dysregulated pathways were obtained from DAVID functional enrichment analysis, based on the abnormal genes identified by meta-analysis. This study demonstrated that cross-GSEA is a useful method for exploring the critical pathways involved CCRCC; however, an individual dataset with a small sample may introduce bias. A cross-GSEA based on certain well-designed datasets may be required to further the progress made in this study, following the analysis of its results.

## Introduction

Renal cell carcinoma (RCC) is the most lethal type of cancer in the urinary system. It is a heterogeneous group of malignancies, and clear cell, papillary and chromophobe RCC are the major subtypes (1). In addition, clear cell RCC (CCRCC) is the most common type of RCC, constituting >80% of cases, and accounts for 2–3% of all adult malignant neoplasms. There were 58,240 new diagnoses of RCC or cancer of the renal pelvis and 13,040 mortalities due to the disease in 2010 in the United States. Furthermore, RCC accounted for 3% of all malignant tumors in adults and 85% of all primary malignant kidney tumors (2). RCC often presents as a metastatic disease and there are no effective treatments for it. This cancer exhibits resistance to chemotherapy and radiation and <10% of patients suffering from the metastatic disease survive five years subsequent to diagnosis. At present, there are no clinically validated adjuvant therapies for patients with CCRCC who are at a high risk of relapse following surgical resection (3). Overall, 40% of patients with RCC succumb to this disease (4). Certain molecular mechanisms have been revealed in RCC, such as von Hipple-Lindau (VHL) gene mutation. This appears to be responsible for ~60% of the cases of sporadic CCRCC (5). In hereditary papillary renal carcinoma, an oncogene, mesenchymal-epithelial transition factor (MET), has been revealed to be mutated, although the incidence of c-MET mutations is low in sporadic papillary RCC. However, to date, no theory has been able to explain all the cases of CCRCC, and there are no effective tumor markers that may be used for diagnostic, prognostic and treatment purposes in CCRCC. To develop novel therapeutic interventions for CCRCC an improved understanding of the pathogenesis is required, since traditional single-factor analysis for tumor pathogenesis is not effective. In order to obtain an enhanced understanding of the potential ‘cross-talks’ between dysregulated genes and proteins, there has been particular focus on undertaking a more ‘global’ analysis (6). Gene set enrichment analysis (GSEA), based on gene expression profiling, has been performed in CCRCC and has identified certain dysregulated gene sets in patients with G1 and G3 RCC (7).

At present, gene expression profiling between tumor tissue and normal tissue at the RNA level has been used in a previous study of RCC (6). A number of differentially expressed genes have been identified in these studies; however, few studies have been performed based on pathway analysis. While it is not difficult to obtain the gene expression profiles, the extraction of biological insight from such information remains a major challenge (8). Genome analysis provides a large quantity of information concerning molecules that are involved in disease pathogenesis and may be used to interpret diseases. Pathway analysis is a frequently used analytical tool. GSEA, which was introduced by Mootha *et al*(9), is well known and extensively used in gene set analysis. GSEA is a computational method that identifies pre-defined gene sets that exhibit significant differences in expression between samples from normal individuals and patients. Following its introduction, the methodology of GSEA was subsequently improved by Subramanian *et al*(8). This modified approach aimed to identify the significant differences in the expression of groups or pathways rather than individual genes. In view of the slight nature of the functions of individual genes, GSEA is more efficient than individual gene analysis for the study of complex diseases (10). Based on the study of a group of associated genes, investigators are likely to have an improved chance of determining the pivotal functional processes responsible for biological phenomena. GSEA in a single dataset is unlikely to identify the significant pathways and there is likely to be a poor concordance. In order to obtain improved data convergence and provide a systematic insight into the pathways altered during CCRCC pathogenesis, standardized microarray preprocessing, GSEA and DAVID functional enrichment analysis were performed in the present study.

## Materials and methods

### Selection and obtainment of datasets

The Gene Expression Omnibus (GEO) (http://www.ncbi.nlm.nih.gov/geo/) and ArrayExpress (http://www.ebi.ac.uk/arrayexpress/) were searched for gene expression profiling datasets associated with CCRCC, using the keywords ‘renal cell carcinoma’ up to March 25, 2013. The inclusion criteria for the datasets were as follows: i) The dataset was genome-wide; ii) samples included patients with CCRCC and normal controls; iii) complete microarray raw or normalized datasets were available.

In addition, seven public gene expression datasets that concurred with our inclusion criteria were included in our study to assess the transcripts of CCRCC on a genome-wide basis. In datasets GSE15641 and GSE11151, there were other types of pathological samples besides CCRCC and normal tissue; in these instances, only the CCRCC and normal tissue samples were retained. In dataset GSE16449, samples from GSM413237 to GSM413270 had the same probe number of 33998; however, the other 35 samples had a different identical probe number and different data precessing, and were excluded. In dataset GSE12606, the samples comprised three CCRCC tissues, including autologous normal tissue and autologous metastatic tissue; the autologous metastatic samples were excluded. Two platforms, HG-U133A and HG-U133B, were applied in datasets GSE781 and GSE6344, however, only the expression profiles in HG-U133A were included in the present study. The basic information with regard to these datasets, such as the microarray platform, experimental design, probe numbers and sample size, are listed in Table I.

### Standardized microarray preprocessing of the datasets

The datasets were processed with an improved software package based on version 2.4.0 of Bioconductor (18) and R version 2.10.1 (19). Dataset GSE16449 comprised normalized data and was retained. Each Affymetrix dataset was computed using the Robust Multichip Averaging (RMA) algorithm in the Affy package (20,21). Genes were excluded from further analysis if it was not possible to map them to any Kyoto Encyclopedia of Genes and Genomes (KEGG) pathway (http://www.genome.jp/kegg/pathway.html). Interquartile ranges (IQRs) were used to describe the level of variability and the genes with an IQR value of <0.5 were excluded. The probe-set with the largest variability was retained when several probe-sets hit one gene.

### GSEA for pathways

GSEA was performed separately in each dataset in the Category package (version 2.10.1) (19). Pathways were excluded if <10 genes were identified to be dysregulated. The t-statistic mean of the genes was computed in each pathway. Using a permutation test with 1,000 times, the significantly changed pathways were identified with a P-value of ≤0.05.

### Meta-analysis and DAVID functional enrichment analysis

A Student’s t-test comparing the CCRCC and normal samples for every probe was performed independently in all of the six Affymetrix datasets using Excel 2007, and probes with a P-value <0.05 were considered to be significantly different. In all included datasets, with the exception of dataset GSE16449 with the Agilent platform, other datasets were checked with the Affymetrix platform. In order to keep the data uniform, we only do meta-analysis on the datasets with Affymetrix platform. Following the t-test, the χ^2^ square value of each selected probe was calculated using the formula: χ^2^=∑(−2 log_e_*p*^i^) (22). The probes with χ^2^ values <0.05 were retained and used to obtain KEGG pathways from DAVID Bioinformatics Resources 6.7 (http://david.abcc.ncifcrf.gov/).

## Results

### Re-analysis of each dataset to obtain the dysregulated pathways

The seven datasets included 110 CCRCC and 73 normal samples. The common method of GSEA was implemented for all of the seven datasets, respectively. In dataset GSE12606, GSEA did not identify any dysregulated pathways. This result may have been due to the small sample size of GSE12606 (three cases and three controls). For the remaining six datasets, significantly abnormal pathways were identified using GSEA, based on the permutation P-values. The results are described in Table II.

### Common pathways identified by GSEA

The results of the GSEA were compared and it was revealed that 17 down- and 12 upregulated pathways coexisted in six datasets. The majority of the downregulated pathways were associated with carbohydrate, lipid and amino acid metabolism, while others were involved in organismal systems or cellular processes. The names of these pathways are listed in Table III. A comparison of the dysregulated pathways, including the up- and downregulated pathways, revealed that certain pathways were upregulated in one or more datasets while being downregulated in another or others.

### Dysregulated pathways identified by DAVID functional enrichment analysis

The χ^2^ value for 3,149 probes <0.05 was calculated using the following formula: χ^2^=∑(−2 log_e_*p*^i^). The IDs of these probes were all used in the DAVID enrichment analysis. A total of 1,023 probes (genes) led to further pathway analysis and 32 dysregulated pathways were identified. The abnormal pathways and the genes involved are listed in Table IV.

## Discussion

CCRCC is a frequently occurring type of cancer that presents numerous challenges. Although a number of preceding studies have attempted to identify the pathogenesis and mechanisms behind this type of cancer, none of the existing theories are able to explain all the cases of CCRCC. Genome-wide expression chips are powerful tools that enable the comprehensive identification of abnormal gene families or pathways present in relevant disease states. Thus, biologically relevant inferences may be reproducible in different studies. For single-gene analysis, examining the same biological condition using different statistical methods and datasets may lead to significant discrepancies (23). Compared with gene expression obtained from different datasets, pathway analysis applied to different datasets yields consistent results and diminishes large discrepancies. Therefore, pathway analysis is able to highlight genes weakly connected to the phenotype that may be difficult to detect in classical univariate statistics.

In our study, GSEA was performed using seven independent publicly available gene expression datasets and the six of these datasets that utilized the Affymetrix platform underwent a meta-analysis and pathway analysis using DAVID functional enrichment analysis. A cross-study based on GSEA was performed to identify critical pathways and to obtain a deeper understand of the common mechanisms involved in CRCC. The results suggested that the majority of the dysregulated pathways were consistent in different studies. Many of the dysregulated pathways identified in the present study have already been indicated to be involved in CCRCC or other types of cancer. In the following paragraphs, a number of the dysregulated pathways and hypotheses regarding the importance of these pathways in CCRCC, based on the functional classification, are discussed.

As shown in Table III, a number of common downregulated pathways were identified by GSEA, including the citrate cycle (TCA cycle) and its associated pathways. Previous studies have demonstrated that the metabolism of a variety of nutrients is dysregulated in certain types of RCC. VHL, MET, folliculin (FLCN), tuberous sclerosis 1 (TSC1), TSC2, fumarate hydratase (FH) and succinate dehydrogenase (SDH) are known as renal cancer genes, and all are involved in pathways that respond to metabolic stress or nutrient stimulation. Individuals who harbor mutations in any of these genes have an increased risk of developing RCC. It has been suggested that RCC may be regarded as a metabolic disease (24). When the current study focused on CCRCC, the TCA cycle pathway and a number of the pathways associated with it were identified as being downregulated. FH and SDH genes (SDHA, SDHC and SDHD) were also identified as having a low expression levels. The FH and SDH genes encode mitochondrial tricarboxylic acid cycle enzymes that are essential in energy metabolism. With deficient FH and SDH expression, the process of the TCA cycle is inhibited and oxidative phosphorylation is hindered. Previous studies have shown that certain FH-deficient kidney tumor cell lines exhibit significantly impaired oxidative phosphorylation (25,26) and are glucose-dependent (25–27). It has been demonstrated that the glucose-mediated generation of cellular reactive oxygen species in an FH-deficient kidney cancer cell line results in the stabilization of hypoxia-inducible factor (HiF) 1-α (27).

Similar to FH, mutations in three of the four SDH genes (SDHB, SDHC and SDHD) have been shown to be involved in familial paraganglioma and familial pheochromocytoma (28,29). Deficient expression of SDH genes severely impairs oxidative phosphorylation and results in SDH-deficient kidney tumors showing glucose dependence. SDH-deficient kidney tumors are likely to be as sensitive to glucose as FH-deficient tumors and targeting the vasculature or glucose transport in these tumors may provide a novel approach to disrupt the fundamental metabolic machinery of these aggressive cancers. Mutations of FH and SDH cause the inactivation of prolyl hydroxylase (PHD), resulting in the stabilization of HiFs and driving the transcriptional activation of genes supporting tumor growth, neovascularization, invasion and metastasis. In the present study, FH and SDH were revealed to have a low expression level in CCRCC. Therefore, we propose that the downregulation of oxidative phosphorylation, the TCA cycle pathway and its associated pathways may have a critical role in the generation and development of CCRCC. The abnormal metabolism of the upstream nutrients of the TCA cycle may therefore be attributed to the blocked TCA cycle.

The tight junction pathway and its associated genes are also indicated to be important in CCRCC. Tight junctions are composed of occludin, claudins and junctional adhesion molecules. Together with adherens junctions and desmosomes, they form the apical junctional complex in epithelial and endothelial cellular sheets. Tight junctions are essential for the tight sealing of the cellular sheets, thus controlling paracellular ion flux and maintaining tissue homeostasis (30). Due to the fact that tight junction proteins are able to recruit signaling proteins (31), these junctions have been assumed to be involved in the regulation of proliferation, differentiation and other cellular functions. There is a generally accepted view that tumorigenesis is accompanied by a disruption of tight junctions. Furthermore, the disruption of tight junctions may be important in the loss of cohesion, invasiveness and lack of differentiation observed in cancer cells. Consistent with this idea, several previous studies have revealed that the expression of occludin and claudins are altered in a number of types of cancer. Occludin was shown to be frequently downregulated in gastrointestinal tumors (32) and a decreased expression of claudin-1 was observed in breast (33,34) and colon cancer (35). The expression pattern of claudins is tissue-specific and it has been demonstrated that claudin-3 (transcript of CLDN3) and/or claudin-4 (transcript of CLDN4 ) are expressed in normal renal tissue (36). To the best of our knowledge, there have been no studies investigating the differential expression of CLDN3 and CLDN4 in normal renal and RCC tissues. When CCRCC tissues were compared with normal renal tissues using GSEA, the tight junction pathway was revealed to be downregulated in CCRCC tissues in different studies. Furthermore, CLDN3, CLDN4, CLDN10, junctional adhesion molecule 2 (JAM2) and JAM3, whose transcripts contribute to the formation of tight junctions, showed decreased expression in CCRCC tissue. Following the review of previous studies, Morin (37) considered that it was possible that the loss of claudins and other tight junction proteins in cancer was responsible for the loss of cell adhesion, an important step in the progression of cancer to metastasis (37). Therefore, the destruction of tight junctions may be important in the development of CCRCC.

A further factor that was considered to be important in the pathogenesis of CRCC was the downregulation of retinol metabolism and its associated genes. It is generally recognized that retinol and its metabolites have significant effects in cell differentiation and apoptosis. In the present study, GSEA revealed that retinol metabolism was downregulated when comparing CCRCC tissues with normal renal tissues, and a number of genes included in this pathway were observed to have a low level of expression, such as alcohol dehydrogenase 1A (ADH1A), ADH1B, ADH1C and cytochrome P450s (CYPs). The inhibition of retinol metabolism results in a lack of its metabolite and, in this case, the blockage of the differentiation and apoptosis of cells. A number of the metabolites are currently widely used in the treatment of several types of cancer. The most notable is the use of all-*trans* retinoic acid in the treatment of acute promyelocytic leukemia (38). Furthermore, several studies have demonstrated that one of the metabolites of retinol, 13-*cis*-retinoic acid, showed improved efficacy in the treatment of RCC when combined with other antitumor drugs (39–44). The results of the current study further indicated that the dysregulated metabolism of retinol may be important in CCRCC. In order to further reveal the role of downregulated retinol metabolism in CCRCC, additional studies focusing on these abnormal genes and the affected metabolites are required. The determination of whether retinol or its metabolites exert therapeutic effects in the treatment of CCRCC may be of benefit.

In addition to downregulated pathways, GSEA also identified a number of pathways that were commonly upregulated. Appearing as solid malignant neoplasms in the human body, RCC has been demonstrated to be immunogenic. A number of immune cells have been isolated from RCC, including natural killer (NK) cells, cytotoxic T cells with specificity for autologous tumor cells, helper T cells and dendritic cells that express interleukin (IL)-1 and IL-2 and function as antigen-presenting cells (45–48). When RCC appears as an antigen in the human body, immune activity is induced, leading to a series of enhanced immunization activities. Nine pathways, which were associated with the immune system or immune system diseases and which co-existed in six independent studies, were identified to be upregulated when we compared CCRCC tissue with normal renal tissue using GSEA. Antigen processing and presentation, cytokine-cytokine receptor interaction and its associated pathways, including the chemokine signaling pathway, and the Jak-STAT signaling pathway were revealed to be upregulated in the present study.

When focusing on the chemokine signaling pathway, it was revealed that many genes involved in this pathway were overexpressed. Consistent with a number of previous studies (49,50), CXCR4, a gene with a transcript known as fusin, and its ligand gene CXCL12, whose transcript is known as stromal cell-derived factor-1α (SDF-1α), were demonstrated to be overexpressed in CCRCC in our study. CXCR4 is considered to be the major chemokine receptor expressed on cancer cells (51–53) and its expression is required for tumor metastasis to other organs; CXCR4 activation by CXCL12 induces the migration of neoplastic cells (54). The strong expression of CXCR4 has been indicated to correlate with the metastasis of RCC (55). The expression of CXCR4 and SDF-1α is negatively regulated by VHL (49), i.e. a deficiency in VHL expression results in the strong expression of CXCR4 and CXCL12. It has been proposed that blocking signaling through this chemokine receptor may inhibit micrometastases in RCC and result in improved outcomes (56). Therefore, the in-depth study of the adjustment mechanisms of CXCR4 expression may be beneficial in the development of novel treatments for metastatic RCC.

In mammals, the Jak-STAT pathway is the principal signaling mechanism for numerous cytokines and growth factors. These signals induce proliferation or cell fate determination and are crucial to the proper growth and development of mammalian tissues. Too strong or too weak activity of this signaling pathway leads to severe consequences. One of these cytokines, interferon-α (IFN-α) is widely used in the therapy of several neoplasms, including RCC. IFN-α exerts its effects by activating the Jak-STAT signaling pathway and subsequently inducing target gene transcription (57). Shang *et al*(58) demonstrated that the defective ability of Jak-STAT in RCC may result in IFN-α resistance (58). In the present study, the Jak-STAT signaling pathway was shown to be upregulated in CCRCC tissue. This was inconsistent with the fact that a substantial proportion of patients with RCC fail to respond to IFN-α treatment. The mechanisms of IFN resistance in RCC have not yet been fully elucidated. Further study for an in-depth understanding of this mechanism may be beneficial for the development of novel treatments.

NK cells are one type of infiltrating immune cell present in RCC and the NK cell-mediated cytotoxicity pathway was shown to exhibit strong activity in CCRCC in the present study. Enhancing CCRCC cell sensitivity to the cytotoxicity of NK cells may be an efficient treatment for CCRCC. Several studies concerning new treatments for RCC based on the toxic effects of NK cells have been performed (59,60).

As malignant neoplasms appear in the human body, CCRCC tissue shows as a self-antigen and induces an autoimmune response. Human leukocyte antigens (HLAs) are key components of the major histocompatibility complex (MHC), which is involved in immune cell signaling processes. Therefore, the overexpression of HLA-B, HLA-C and a number of other HLA alleles, as well as the upregulation of certain immune system disease pathways in CCRCC, may be attributed to the self-antigenicity of CCRCC.

The present study also compared the common dysregulated pathways identified by GSEA with the dysregulated pathways obtained from DAVID enrichment analysis. The TCA cycle pathway, numerous nutrient metabolic pathways and the tight junction pathway were shown to be dysregulated in DAVID functional enrichment analysis, in addition to GSEA, based on the abnormal genes identified by meta-analysis. However,, a number of the dysregulated pathways identified using DAVID functional enrichment analysis were not consistent with the cross-GSEA. In this discussion, only the abnormal pathways co-existing in the six datasets have been regarded as significant and discussed. When we reviewed the design of every dataset included in our study, however, certain datasets had small control samples and it was difficult to identify the significant genes. This indicated that a dataset with a small sample size may lead to bias. We also analyzed the dysregulated pathways co-existing in five of the six datasets. There were 17 down- and 12 upregulated pathways co-existing in five of the CCRCC datasets. When compared with the dysregulated pathways in the meta-analysis, nitrogen metabolism, DNA replication, the peroxisome proliferator-activated receptor (PPAR) signaling pathway, nucleotide excision repair, cell cycle, cell adhesion molecules (CAMs), the Toll-like receptor signaling pathway and *Vibrio cholerae* infection were reduplicated. This result further indicated that the poor design of the individual dataset may lead to large bias in a cross-GSEA.

Numerous treatments have been developed based on the current understanding of RCC; however, none of the existing therapies are universally valid. The treatment of metastatic RCC remains a significant challenge. More effectual studies are required to reveal the pathogenesis and progress of RCC. The current study demonstrated that a cross-GSEA, based on a number of well-designed datasets, is likely to improve the consistency of the understanding of RCC. Additional cross-GSEAs may further the progress made in this study.

In conclusion, cross-GSEA was used to identify the critical pathways involved in RCC using gene expression datasets of CCRCC. A number of pathways that were associated with RCC or CCRCC were identified as being significant, and the understanding of RCC has been improved. The downregulation of the TCA cycle pathway, retinol metabolism and the tight junction pathway may be critical in the occurrence of CCRCC. In addition, the upregulation of the chemokine signaling pathway and its associated pathways may contribute to the metastasis of CCRCC; however, this is the focus of immunotherapy. A further cross-GSEA based on a number of well-designed datasets may provide a valuable contribution to the progress made in the current study, following the analysis of its results.

## Figures and Tables

**Table I:** Datasets information included in this study.

First author or contributor	Platform	GEO accession	Experimental design	Probes	Number of samples	

					Disease	Normal
Lenburg (11)	HG-U133A	GSE781	Unpaired tissues	22K	12	5
Jones (12)	HG-U133A	GSE15641	Unpaired tissues	22K	32	23
Wang (13)	HG-U133 Plus 2	GSE14762	Unpaired tissues	54K	10	12
Yusenko (14)	HG-U133 Plus 2	GSE11151	Unpaired tissues	54K	26	3
Gumz (15)	HG-U133A	GSE6344	Paired tissues	22K	10	10
Brannon (16)	G4112F	GSE16449	Paired tissues	41K	17	17
Stickel (17)	HG-U133 Plus 2	GSE12606	Paired tissues	54K	3	3

Paired: Clear cell renal cell carcinoma (CCRCC) tissue was compared with the corresponding normal renal tissue from the same patient. Unpaired: CCRCC tissue from patients with CCRCC was compared with the renal tissue from a normal kidney. GEO, Gene Expression Omnibus.

**Table II:** Information summary regarding each dataset used in the re-analysis.

GEO accession	Patient number	Control number	Gene number of preprocessing	Upregulated pathways (P<0.05)	Downregulated pathways (P<0.05)
GSE781	12	5	2292	17	41
GSE15641	32	23	2325	68	31
GSE14762	10	12	2754	41	52
GSE11151	26	3	2702	88	26
GSE6344	10	10	2287	69	65
GSE16449	17	17	3557	40	96
GSE12606	3	3	2710	0	0

GEO, Gene Expression Omnibus.

**Table III:** Common dysregulated pathways identified by gene set enrichment analysis (GSEA).

Pathway names	Classification	Genes
Upregulated pathways		
Cytokine-cytokine receptor interaction	Signaling molecules and interaction	50
Chemokine signaling pathway	Immune system	42
Antigen processing and presentation	Immune system	30
Jak-STAT signaling pathway	Signal transduction	30
Natural killer cell mediated cytotoxicity	Immune system	33
Type I diabetes mellitus	Metabolic diseases	25
Asthma	Immune system diseases	11
Autoimmune thyroid disease	Immune system diseases	18
Systemic lupus erythematosus	Immune system diseases	44
Allograft rejection	Immune system diseases	18
Graft-versus-host disease	Immune system diseases	15
Primary immunodeficiency	Immune system diseases	11
Downregulated pathways		
Citrate cycle (TCA cycle)	Carbohydrate metabolism	20
Fatty acid metabolism	Lipid metabolism	34
Oxidative phosphorylation	Energy metabolism	28
Alanine, aspartate and glutamate metabolism	Amino acid metabolism	17
Glycine, serine and threonine metabolism	Amino acid metabolism	22
Valine, leucine and isoleucine degradation	Amino acid metabolism	31
Arginine and proline metabolism	Amino acid metabolism	29
Tyrosine metabolism	Amino acid metabolism	14
Tryptophan metabolism	Amino acid metabolism	20
β-alanine metabolism	Metabolism of other amino acids	15
Selenoamino acid metabolism	Metabolism of other amino acids	15
Pyruvate metabolism	Carbohydrate metabolism	20
Propanoate metabolism	Carbohydrate metabolism	21
Butanoate metabolism	Carbohydrate metabolism	23
Retinol metabolism	Metabolism of cofactors and vitamins	17
Cardiac muscle contraction	Circulatory system	10
Tight junction	Cell communication	26

Genes: Number of common genes included in the pathway.

**Table IV:** Dysregulated pathways identified by DAVID functional pathway analysis based on the abnormal gene.

Pathway entry	Pathway names	Counts	Genes
00010	Glycolysis/gluconeogenesis	22	PGK1, PKLR, PCK2, ALDH2, PKLR, DLAT, ADH5, ADH5P4, LOC652797, PKM2, G6PC, ALDH7A1, HK1, FBP1, PFKP, ADH1C, ADH1B, ADH1A, ALDH7A1, PDHA1, ALDOC, ADH1C, ADH1B, ADH1A, ENO2, PGK1, PGAM2, DLD, HK2P1, HK2, PDHB, ALDH9A1, TPI1, TPI1P1
00020	Citrate cycle (TCA cycle)	14	FH, MDH1, PCK2, ACO2, OGDH, PDHA1, DLAT, IDH2, LOC100130320, SDHD, SDHB, DLD, SUCLG1, PDHB, IDH1
00062	Fatty acid elongation in mitochondria	5	ECHS1, HADHB, ACAA2, LOC648603, PPT1, PPT2
00071	Fatty acid metabolism	17	ACAT1, GCDH, ACSL1, CPT1A, EHHADH, ALDH2, ALDH7A1, ADH1C, ADH1B, ADH1A, ECHS1, ADH1C, ADH1B, ADH1A, HADHB, ACAA2, LOC648603, ADH5, ADH5P4, ACADM, ACSL3, ACADSB, ALDH9A1, ALDH7A1, ACSL6
00260	Glycine, serine and threonine metabolism	15	SDS, AMT, SHMT2, GLDC, AGXT, PHGDH, CTH, SRR, MAOA, BHMT, AGXT, GATM, DAO, GCAT, DLD, GCAT, SARDH
00280	Valine, leucine and isoleucine degradation	25	MUT, ACAT1, HMGCS2, EHHADH, ALDH2, MCCC2, MCCC1, ECHS1, HMGCL, HADHB, ACAA2, LOC648603, PCCA, ACADSB, ALDH7A1, AUH, ABAT, ALDH7A1, ABAT, HSD17B10, BCKDHB, OXCT1, AOX1, ALDH6A1, BCAT1, ACADM, DLD, ALDH9A1
00330	Arginine and proline metabolism	18	P4HA2, ARG2, GLS, ALDH4A1, CKMT2, ALDH2, ALDH7A1, MAOA, GOT2, NOS1, SRM, OAT, CKB, GATM, DAO, ACY1, ALDH9A1, GOT1, ALDH7A1
00380	Tryptophan metabolism	14	CAT, ACAT1, DDC, GCDH, EHHADH, OGDH, WARS, LDH2, ALDH7A1, MAOA, ECHS1, IDO1, AOX1, ALDH9A1, ALDH7A1
00410	β-alanine metabolism	9	ECHS1, ABAT, GAD1, ACADM, EHHADH, ALDH2, ABAT, ALDH7A1, ALDH9A1, SRM, ALDH7A1
00620	Pyruvate metabolism	17	ACACB, PKLR, ACACB, ACACA, MDH1, PCK2, ACAT1, ACACB, ME2, ALDH2, ALDH7A1, ME1, PDHA1, PKLR, DLAT, ME2, DLD, ME3, LOC652797, PKM2, PDHB, ALDH9A1, ALDH7A1
00640	Propanoate metabolism	15	ACACB, ACACB, ACACA, ACAT1, ACACB, MUT, EHHADH, ALDH2, ABAT, ALDH7A1, ECHS1, ABAT, ALDH6A1, PCCA, ACADM, SUCLG1, ACSS3, ALDH9A1, ALDH7A1
00650	Butanoate metabolism	17	GAD1, ACAT1, HMGCS2, EHHADH, L2HGDH, ALDH2, ABAT, ALDH7A1, PDHA1, ACSM3, ECHS1, ABAT, ACSM2A, HMGCL, OXCT1, ALDH5A1, PDHB, ALDH9A1, ALDH7A1
00670	One carbon pool by folate	9	MTHFD2, MTHFS, MTHFR, AMT, SHMT2, TYMS, GART, ALDH1L1, ALDH1L1, MTHFD1
00680	Methane metabolism	4	CAT, MTHFR, SHMT2, ADH5, ADH5P4
00910	Nitrogen metabolism	9	CA6, AMT, GLS, CA2, CA4, CTH, CA12, CA9, CA8
03030	DNA replication	17	POLD2, RFC2, PRIM1, PCNA, POLE2, POLA1, RFC4, MCM5, RFC5, RPA2, RPA1, RNASEH2A, MCM3, RPA4, MCM6, RNASEH2B, LIG1
03320	PPAR signaling pathway	22	NR1H3, PCK2, FABP7, HMGCS2, PDPK1, ADIPOQ, ACSL1, ACOX2, CPT1A, EHHADH, GK3P, GK, ME1, PPARA, OLR1, FABP2, SLC27A2, ACADM, FABP3, FABP6, AQP7, ACSL3, ACSL6
03420	Nucleotide excision repair	16	POLD2, RFC2, RBX1, PCNA, POLE2, RFC4, RFC5, RPA2, ERCC1, RPA1, ERCC5, ERCC3, RPA4, DDB2, XPC, LIG1
03430	Mismatch repair	11	RFC5, POLD2, RPA2, RFC2, RPA1, PCNA, MSH6, RPA4, MSH2, RFC4, LIG1
04012	ErbB signaling pathway	26	ERBB4, CRKL, MAPK10, HRAS, CAMK2B, PLCG2, MTOR, PIK3CG, CAMK2G, AREGB, AREG, MAPK1, RPS6KB1, PRKCA, EIF4EBP1, CAMK2A, MAPK9, NCK2, TGFA, PIK3R5, PIK3CD, PRKCA, PAK2, PAK6, PAK4, CDKN1B, EGF, CBLB, ERBB4, PRKCA, CAMK2G, PRKCA
04110	Cell cycle	36	STAG1, TP53, CCNB2, LOC651610, ATM, RBL1, CDC14B, PCNA, RB1, YWHAH, TTK, CCNE2, CDC7, MAD1L1, BUB1B, MCM5, CCNA2, CDKN2C, CDK2, CDC27, ANAPC1, LOC100133982, LOC100133898, CCNA2, ORC5L, CDK1, CDC27, CDKN2A, RBX1, CCND3, CHEK1, BUB1, CDC14B, CDKN1B, GADD45A, CDKN1C, CDKN2A, CND1, TGFB2, TGFB1, MCM3, SMAD4, STAG1, MCM6
04210	Apoptosis	25	LOC651610, ATM, TP53, FADD, ENDOG, TRADD, EXOG, PIK3CG, PRKACG, PRKAR2A, CAPN2, CHP2, PRKAR2B, AIFM1, IRAK4, CSF2RB, RIPK1, PIK3R5, PIK3CD, DFFA, CYCS, TNFRSF10B, MYD88, CASP3, BIRC2, BID
04514	Cell adhesion molecules (CAMs)	39	CD4, CD40, MPZL1, CDH2, CD40, CD8A, HLA-F, HLA-DRA, CD86, HLA-DPA1, SIGLEC1, ITGA4, CDH15, CLDN10, PTPRC, CADM1, NFASC, SELPLG, CD2, SDC4, CDH4, SDC3, CDH5, HLA-E, CLDN16, PTPRM, CLDN14, OCLN, LOC647859, PECAM1, PVRL1, PECAM1, HLA-DPA1, CLDN6, LOC284620, NFASC, HLA-DOA, ITGA4, SELL, HLA-DMB, CLDN15, NLGN1, CD40, ICAM3, L1CAM, CLDN8, CDH3
04530	Tight junction	36	MYH8, PRKCH, PRKCZ, HRAS, TJP3, EPB41L1, GNAI3, LLGL1, MLLT4, LOC730031, MYH10, MPDZ, MYL10, TJP1, MPP5, PRKCA, TJP2, CLDN10, PARD6A, INADL, GNAI1, MAGI1, CNKSR3, RRAS, SPTAN1, MYH8, EPB41L1, ACTN1, PRKCA, EXOC3, HCLS1, CLDN16, MAGI2, CLDN14, OCLN, LOC647859, MYH14, MYH10, PRKCQ, CLDN6, LOC284620, PRKCA, CLDN15, TJP3, PRKCE, MYH14, CLDN8, PRKCA, MAGI1, CNKSR3
04610	Complement and coagulation cascades	20	VWF, C1QB, C7, C2, C5, F12, BDKRB2, KNG1, CR1, PROC, KLKB1, SERPINA5, C3AR1, PLG, CFB, CFH, MASP2, KNG1, LOC653879, LOC100133511, C3, C1QA, C5AR1
04620	Toll-like receptor signaling pathway	28	CXCL11, CD40, TLR8, FADD, TLR2, MAPK10, IRF3, CCL5, TBK1, CD40, TLR3, PIK3CG, CD86, CXCL10, MAPK1, TLR7, IRAK4, MAPK9, RIPK1, PIK3R5, CXCL9, PIK3CD, CD14, CCL5, CCL4, LY96, MYD88, TLR1, CD40, MAPK14, TICAM1
04910	Insulin signaling pathway	42	PKLR, ACACA, ACACB, CRKL, PCK2, MAPK10, ACACB, PRKCZ, IRS1, CALM3, CALM2, CALM1, HRAS, PRKAB1, PPP1R3C, CALM3, CALM2, CALM1, MTOR, PRKACG, PIK3CG, PKLR, PRKAR2A, RHOQP2, RHOQ, MAPK1, INS-IGF2, INS, IGF2, RPS6KB1, PRKAR2B, EIF4EBP1, PRKAA2, MAPK9, SLC2A4, G6PC, HK1, PIK3R5, ACACB, FBP1, PIK3CD, PDPK1, PYGL, GYS2, PTPN1, CBLB, PPP1CC, SREBF1, PPARGC1A, SOCS2, HK2P1, HK2, PHKA2, PPP1R3D
04920	Adipocytokine signaling pathway	23	NFKBIE, ACACB, MAPK10, ACACB, PCK2, ADIPOQ, IRS1, ACSL1, PRKAB1, NPY, TRADD, PPARA, MTOR, PRKAA2, MAPK9, SLC2A4, G6PC, ACACB, SLC2A1, CPT1A, PRKCQ, TNFRSF1B, PPARGC1A, ACSL3, ACSL6
04930	Type II diabetes mellitus	18	PIK3R5, HK1, PKLR, PIK3CD, MAPK10, PRKCZ, ADIPOQ, IRS1, PIK3CG, MTOR, PKLR, INS-IGF2, INS, IGF2, MAPK1, SOCS2, HK2P1, HK2, LOC652797, PKM2, MAPK9, SLC2A4, PRKCE
04960	Aldosterone-regulated sodium reabsorption	16	PIK3R5, PIK3CD, SCNN1B, PRKCA, PDPK1, IRS1, KCNJ1, PIK3CG, NR3C2, ATP1B3, NEDD4L, INS-IGF2, INS, IGF2, MAPK1, PRKCA, PRKCA, ATP1B2, HSD11B2, SCNN1A, PRKCA
05110	*Vibrio cholerae* infection	17	ATP6V1E1, PRKCA, GNAS, ATP6V1B1, GNAS, ATP6V1G2, ATP6V0A4, PLCG2, PRKACG, KDELR3, ATP6V1A, ATP6V0C, TJP1, SEC61G, PRKCA, TJP2, CFTR, PRKCA, ATP6V1D, PDIA4, PRKCA
05214	Glioma	20	TP53, HRAS, CALM3, CALM2, CALM1, CAMK2B, RB1, PLCG2, CALM3, CALM2, CALM1, PIK3CG, MTOR, PDGFRA, CAMK2G, IGF1R, MAPK1, PRKCA, CAMK2A, TGFA, PIK3R5, PIK3CD, CDKN2A, PRKCA, EGF, CDKN2A, CCND1, PRKCA, CAMK2G, PRKCA

Counts: Number of the abnormal genes included in related pathway. Genes: Office name of the abnormal genes, some genes have several names and all of these were listed. PPAR, peroxisome proliferator-activated receptor.
